# Translation, adaptation, and validation of a Moroccan version of the H-SCALE (Hypertension Self-Care Activity Level effects) for hypertensive patients

**DOI:** 10.1186/s12875-026-03400-8

**Published:** 2026-06-04

**Authors:** Manar Aarrad, Imane Barakat, Saad El Madani, Noureddine El Khoudri, Mohamed Chahboune, Aya Ikhelk, Imane Kabbach, Ouiam Yammad, Mohamed Hilal, Fatimazahra Laamiri

**Affiliations:** 1 Health Sciences and Techniques Laboratory, Higher Institute of Health Sciences, Hassan First University, District, 26000 Settat, Morocco; 2 Higher Institute of Nursing Professions and Health Techniques of Marrakech, Marrakech, Morocco

**Keywords:** H-SCALE, Hypertension, Morocco, Psychometric validation, Translation and cross-cultural adaptation

## Abstract

**Background:**

Hypertension is the leading cause of premature death worldwide and the primary risk factor for cardiovascular disease. Its management relies mainly on self-management practices by hypertensive patients. The Hypertension Self-Care Activity Level effects scale (H-SCALE) is one of the most widely used tools for assessing these practices. The objective of this study is to translate this tool into Moroccan dialect and to study the psychometric properties of its Moroccan version. It also aims to assess adherence to hypertension self-care behaviors using the adapted tool.

**Materials and methods:**

A study was conducted in four primary health care centers in Settat city. The validation process included forward–backward translation with cross-cultural adaptation, assessment of content validity by six experts, face validity testing among 30 hypertensive patients, and psychometric evaluation in an independent sample of 120 patients. The questionnaire was administered through face-to-face interviews. Construct validity was assessed using exploratory factor analysis, while reliability was evaluated using Cronbach’s alpha and the intraclass correlation coefficient (ICC). Sociodemographic and clinical data, as well as anthropometric measurements and blood pressure values, were collected.

**Results:**

The study included 120 hypertensive patients, predominantly female (71.7%), with a mean age of 56.64 ± 11.45 years. Construct validity was supported by exploratory factor analysis (KMO = 0.682; Bartlett’s test *p* < 0.001), revealing a six-factor structure explaining 53% of the total variance. The Moroccan version of the scale demonstrated satisfactory internal consistency (Cronbach’s alpha = 0.79) and good test–retest reliability (ICC = 0.80), with domain-specific coefficients ranging from 0.76 to 0.95. Furthermore, treatment adherence (*p* = 0.01) and weight management scores (*p* < 0.001) were significantly higher among patients with controlled blood pressure.

**Conclusion:**

The Moroccan version of the H-SCALE showed satisfactory reliability and acceptable exploratory construct validity. These findings support its potential use for assessing hypertension self-care behaviors in the Moroccan context.

## Background

High blood pressure is one of the leading causes of death worldwide, responsible for nearly 10.4 million deaths each year [1]. The majority of these deaths are attributed to ischemic or hemorrhagic strokes, with hypertension being the main causal factor [2]. Has become a major public health issue on an international scale, Hypertension is recognized as the leading cardiovascular risk factor and remains the cause of often serious, even fatal, cardiac, vascular, and renal complications [3]. According to the World Health Organization (WHO), 1.28 billion adults aged 30 to 79 have hypertension, two-thirds of whom live in low- and middle-income countries [4]. Morocco is part of this trend, ranking among the countries in the eastern Mediterranean region with high mortality rates linked to non-communicable diseases, estimated at 80%, of which 38% are attributed to cardiovascular diseases [5]. The national survey on risk factors for non-communicable diseases revealed a prevalence of hypertension of 29.3% in Morocco, accounting for 11.70% of expenditure related to long-term conditions covered by compulsory health insurance [6]. Back in 2000, the results of the previous national survey identified advanced age, obesity, smoking, and certain comorbidities as factors associated with hypertension, while highlighting that 87.3% of hypertensive patients remained uncontrolled despite treatment [7]. Since then, several regional studies have confirmed the high prevalence of uncontrolled blood pressure (UBP) and highlighted the importance of lifestyle and dietary measures in the management of this chronic disease [8–10]. These measures are based primarily on the self-care responsibilities of hypertensive patients, including adopting a varied diet rich in fruits and vegetables and low in salt, engaging in regular physical activity (PA), quitting smoking and alcohol, and adhering to the prescribed antihypertensive treatment [11]. However, poor adherence to these self-care activities compromises stability and optimal blood pressure control (BPC) [12]. In this context, the existence of a validated tool for assessing adherence to self-care activities is of major interest. Such a tool would enable healthcare professionals to assess the level of compliance with recommendations, adapt treatment protocols, and improve healthcare services. It would also help patients achieve better BPC by identifying their self-management difficulties and guiding them toward healthier behaviors. The transcultural adaptation of such an instrument is also essential in order to take into account the sociocultural specificities of the Moroccan context.

The aim of our work is therefore to translate, adapt, and validate a tool for assessing adherence to hypertension self-care activities in primary healthcare facilities (PHCF) in Settat city, Morocco. It also aims to assess adherence to key hypertension self-care behaviors, including medication adherence, DASH diet practices, physical activity, weight management, smoking cessation, and alcohol consumption, using the adapted tool, given that, to our knowledge, no previous study has addressed this topic in Morocco.

## Materials & methods

### Description of the study

The study was conducted in four medical PHCF in the province of Settat, which provide care and follow-up for patients with hypertension. It was carried out in four successive phases: (a) Translation, back-translation, and transcultural adaptation of the original scale into a Moroccan dialect version, in accordance with international methodological recommendations [13], (b) assessment of content validity by a panel of six experts [14], from the fields of public health, cardiology, and nursing research, (c) assessment of face validity in a sample of 30 hypertensive patients not included in the final sample [15], (d) assessment of construct validity and reliability in a separate sample of 120 hypertensive patients [16] and finally, (e) evaluation of adherence to hypertension self-care activities. The data collected during the first administration of the questionnaire (test) were used to describe the characteristics of the participants and analyze construct validity. The data from the second administration (retest) were used exclusively to assess test–retest reliability by calculating the intra-class correlation (ICC).

### Description of the items on the (H-SCALE)

The original scale (H-SCALE) developed in 2011 and revised in 2013 by Warren-Findlow et al. [17, 18]. Comprises 31 items divided into six key areas of self-management of hypertension:

- Adherence to treatment (Items 1–3):

This subscale assesses adherence to antihypertensive drugs prescribed over the past seven days. It focuses on three elements: regular intake of the treatment, adherence to a fixed time of intake, and intake of the recommended dosage. Each element is scored from 0 to 7 (number of days/week), for a total score ranging from 0 to 21. A score ≥ 17 indicates good adherence to treatment.

- The quality of the DASH diet (DASH-Q) (Item 4–14):

These 11 items assess adherence to the DASH diet (Dietary Approaches to Stop Hypertension) [19], by exploring the frequency of consumption of fruits and vegetables, potassium-rich foods, whole grains, and restriction of foods high in salt. Each item is rated from 0 to 7 indicating the number of days/week, except for the item relating to the consumption of salty foods, which is coded in reverse. The total score ranges from 0 to 77, with a score ≥ 52 reflecting good adherence to the DASH diet.

- Physical activity (Items 15 −16):

This subscale assesses compliance with international recommendations for regular physical activity. The total score ranges from 0 to 14. A score of ≥ 8 indicates satisfactory adherence to physical activity recommendations.

- Smoking (Items 17–18):

Exposure to tobacco is assessed using two items relating to the frequency of tobacco use and exposure to secondhand smoke over the past seven days. Each item is scored from 0 to 7, for a total score of 0 to 14. A score of 0 corresponds to optimal compliance (total absence of exposure).

- Weight management (Items 19–28):

This subscale, consisting of 10 items, explores practices related to eating behaviors and physical activity aimed at weight loss or maintenance. Responses are rated from 1 (“strongly disagree”) to 5 (“strongly agree”), for a total score between 10 and 50. A score ≥ 40 indicates good adherence to weight management strategies.

- Alcohol consumption (Items 29- 31):

For this subscale, abstinence from alcohol was considered good adherence. Adherence thresholds varied according to gender: consumption of ≤ 2 drinks/day was considered moderate in men (score ≤ 14), while consumption of ≤ 1 drink/day was considered moderate in women (score ≤ 7).

### Scale translation approach

The translation of the scale (H-SCALE) into Moroccan dialect was carried out after obtaining permission from the copyright holder, Dr. Warren-Findlow. In order to ensure conceptual and metrological equivalence with the original version, the translation and cross-cultural adaptation process strictly followed the methodological recommendations of Beaton et al. [13]:

1. Initial translation (forward translation)

Two translators independently produced an initial translation of the questionnaire. The first translator was a professor of nursing sciences with a master's degree and familiarity with the concepts and clinical areas covered by the questionnaire. The second translator was a professor of economics and applied finance with no training in health sciences, ensuring that the translation was not influenced by the scientific content of the questionnaire. The two translated versions were compared and discussed at a meeting attended by a group of bilingual experts from various fields (public health, nursing, research methodology). The aim was to examine the differences, assess clarity and semantic equivalence, and arrive at a consensus version.

2. Back translation

The consensus version was independently back-translated by two Moroccan translators fluent in English: a professor teaching advanced health practices at master's level and a professor teaching integrated preparatory classes in biomedical engineering. Neither translator had access to the original questionnaire in order to avoid any conceptual influence.

3. Synthesis and validation of versions

The back-translations obtained were compared with the original version of the H-SCALE by all of the co-authors. Any conceptual, semantic, or cultural differences were analyzed, discussed, and resolved by consensus between the researchers and translators.

This rigorous process resulted in a pre-final version in Arabic dialect that is faithful to the original version and adapted to the Moroccan sociocultural context.

### Internal content validation (ICV) by experts

The content validity of the Moroccan dialect version of the scale was assessed by a panel of six health experts: three general practitioners who are heads of centers practicing in primary health care facilities and three multi-skilled nurses responsible for the management of chronic diseases in different health centers. The objective of this step was to examine the semantic, conceptual, and cultural equivalence of the translated version in relation to the original questionnaire.

Each expert individually evaluated the 31 items of the H-SCALE based on four criteria commonly recommended for content validation: relevance, clarity, simplicity, and lack of ambiguity, which were rated on a four-point Likert scale: 1 = Criterion not met; 2 = Major modifications needed; 3 = Minor revisions needed; 4 = Criterion fully met (15). A score ≥ 3 was considered to indicate satisfactory adequacy between the translated version and the original version.

### Sampling

Participants were recruited from four primary health care facilities in the province of Settat using a non-probabilistic consecutive sampling approach. All hypertensive patients attending these facilities during the study period who met the eligibility criteria were invited to participate until the target sample size was achieved.

The final sample consisted of 120 hypertensive patients, which is considered acceptable for exploratory psychometric validation studies. Methodological recommendations commonly suggest including at least 100 participants when conducting exploratory factor analysis. Therefore, the sample size was deemed adequate to evaluate the psychometric properties of the adapted scale [20].

Eligible participants were aged 18 years or older, had a confirmed diagnosis of essential hypertension for at least six months, and were receiving antihypertensive treatment. Patients with secondary hypertension, gestational hypertension, or cognitive or mental disorders that could interfere with comprehension of the questionnaire were excluded from the study.

### Data collection

Data on hypertensive patients were collected between May 1^First^ 2025 and July 31, 2025. After obtaining informed consent from each participant, a standardized questionnaire was used to collect socioeconomic data (gender, age, educational level, and monthly income) as well as clinical and therapeutic characteristics, including duration of the disease, family history of hypertension, and comorbidities. Anthropometric measurements were taken using standardized procedures. Weight was measured using electronic scales accurate to 100 g, while height was measured barefoot using a wall-mounted height gauge graduated to the nearest tenth of a centimeter. Body mass index (BMI) was calculated to assess the nutritional status of participants, in accordance with the World Health Organization classification [21]. Systolic and diastolic blood pressure was measured using a validated electronic blood pressure monitor (Microlife BP A2 Basic) equipped with a cuff suitable for adults and obese individuals. Two measurements were taken 2 min apart on the arm with the highest values, and the blood pressure taken was the mean of the two values. Patients were classified in the ‘uncontrolled hypertension’ group if their SBP was greater than or equal to 140 mmHg and/or if their DBP was greater than or equal to 90 mmHg. Measurements were taken under standardized conditions, following the recommendations of the International Society of Hypertension [1]. The translated instrument (H-SCALE-M) was administered to a sample of 120 hypertensive patients through face-to-face interviews. This mode of administration was selected in view of the relatively high rate of illiteracy among participants, in order to ensure an adequate understanding of the questionnaire items and to minimize potential errors associated with self-administration. The questionnaire was administered twice (test–retest) with an interval of 7 to 15 days between the two administrations [22].

## Statistical analysis

The collected data were entered into a data sheet and analyzed using SPSS (Statistical Package for Social Sciences) version 27 software. The normality of the distribution of quantitative variables was studied using the Kolmogorov–Smirnov test. Quantitative variables following a Gaussian distribution (age, SBP, and DBP) were expressed as mean and standard deviation, while qualitative variables were expressed as numbers and percentages.

Construct validity of the adapted scale was examined using exploratory factor analysis (EFA). The suitability of the data for factor analysis was first assessed using the Kaiser–Meyer–Olkin (KMO) measure of sampling adequacy and Bartlett’s test of sphericity. Factor extraction was performed using the principal axis factoring method with oblique rotation (Promax), considering the potential correlation between the underlying dimensions of the scale. Factors with eigenvalues greater than 1 were retained, and items with factor loadings ≥ 0.40 were considered to contribute meaningfully to the factor structure.

The internal consistency of the different subscales of the questionnaire was assessed using Cronbach's alpha coefficient, with a value greater than or equal to 0.70 considered satisfactory. To assess test–retest reliability, all participants completed the questionnaire a second time after an interval of 7 to 15 days. Reliability was quantified using the intra-class correlation coefficient (ICC) [23]. The Student's t-test was used to compare the mean scores between the different groups of patients surveyed. A *p*-value < 0.05 was considered statistically significant.

## Results

I. Translation and Transcultural Adaptation
  1. Pre-test Results and Transcultural Adaptation of the Scale

The final translated version of the H-SCALE-M was pre-tested among 30 patients with hypertension (excluded from the final sample). The sample showed a predominance of female participants (56.66%, *n* = 17), with a mean age of 58.42 ± 10.45 years, and suboptimal blood pressure control in the majority of patients (80%, *n* = 24). Participants were interviewed regarding the clarity and comprehensibility of the different items of the scale. Based on the feedback obtained during the pre-test phase, adjustments were made to certain items to ensure better transcultural adaptation of the questionnaire to the Moroccan context. The modifications introduced are as follows:

- Item 4: Examples of dried fruits have been added to make the question clearer. Some regions use the term « الفاكية» while others use the term « السلعة». Thus, the main dried fruits consumed in Morocco were specified by adding the expression « بحال الكاوكاو، اللوز، الكركاع». “"Such as peanuts, almonds, and walnut”.
- Item 9: The term « عصير الفواكه» has been replaced by « عصير الديسير» “fruit juice” in order to adapt the wording to hypertensive patients with varying levels of education.
- Item 11: the product « tea» (« آتاي») has been removed from the list of products added to milk, as this combination is not commonly recognized in the Moroccan food context.
- Item 24: the products « Milk» and « Tea» (« الحليب أو آتاي») have been added to the list of beverages mentioned, due to their frequent consumption in almost all regions of Morocco.
  2) Results of internal content validation (ICV) by experts

Item 12, which belongs to the DASH diet subscale, was the only item to receive a score below 3. The experts interviewed recommended replacing the term “broccoli” with “cabbage,” a vegetable with similar nutritional characteristics that is more commonly consumed in the Moroccan diet. The overall content validity index of the Moroccan version of the H-SCALE (H-SCALE-M) was 0.95, which was considered very satisfactory.

II. Psychometric validation
  1. Socioeconomic and clinical characteristic and blood pressure measurements

One hundred twenty hypertensive patients were included in the study, of whom 71.70% (*n* = 86) were female. The mean age of participants was 56.64 ± 11.45 years. In addition, more than half (54.20% *n* = 65) were aged ≥ 50 years. The majority of patients were married (79.2%, *n* = 95), illiterate (70%, *n* = 80), and unemployed (82.5%, *n* = 99). Furthermore, 75.8% (*n* = 91) of participants had a low socioeconomic status, with a reported monthly income of less than 2,000 dirhams (MAD/month) (Table 1).

**Table 1:** The socioeconomic characteristics of the hypertensive patients surveyed

Socioeconomic variables	Participants *N* = 120
Age (Year)^α^	56.64 ± 11.45
Age group^β^	
< 40	9 (7.50)
40–50	46 (38.30)
50–60	20 (16.70)
> 60	45 (37.50)
Gender^β^	
Female	86 (71.70)
Male	34 (28.30)
Marital status^β^	
Single	5(4.20)
Married	95(79.20)
widower	19(18.50)
Divorced	1(0.80)
Level of education ^β^	
Illiterate	84(70)
Quranic	18(15)
Primary	12(10)
Secondary	6(5)
Employment status^β^	
Employed	21(17.50)
Unemployed	99(82.50)
Income per household (MAD/Month)^β^	
< 2000	91(75.80)
2000—5000	3(2.50)
> 5000	26(21.70)

*MAD* Moroccan Dirhams

^α^Values are expressed as averages and standard deviations

^β^Values are expressed in numbers and percentages. Employment status was classified as employed or unemployed at the time of the survey

Analysis of the clinical and medical characteristics associated with hypertension in the patients surveyed showed that the majority of them (68.33%, *n* = 82) had been hypertensive for five years or more. More than half of the participants (62.50%, *n* = 75) had a family history of high blood pressure. In addition, 44.20% (*n* = 53) of patients were diabetic, 19.20% (*n* = 23) had dyslipidemia, 17.50% (*n* = 21) had heart disease, and 5% (*n* = 6) had kidney disease. Blood pressure control was suboptimal in 73.30% (*n* = 88) of patients. The mean systolic and diastolic blood pressures were 158.85 ± 20.69 mmHg and 88.63 ± 11.51 mmHg, respectively (Table 2).

**Table 2:** Clinical and medical characteristics of hypertensive patients

Clinical and medical characteristics	Participants *N* = 120
Duration of hypertension^β^	
< 5 Year	38 (31.66)
5–10 Year	36 (30)
≥ 10 Year	46 (38.33)
Family history of hypertension^β^	
Yes	75 (62.50)
No	45 (37.50)
Comorbidities^β^	
Diabetes	53 (44.20)
Dyslipidemia	23 (19.20)
Heart disease	21 (17.50)
Kidney disease	6 (5.00)
Current tobacco consumption^β^	
Yes	6 (5.00)
No	114 (95)
alcohol consumption^β^	
No	120 (100)
Blood pressure controle^β^	
Yes	32 (26.70)
No	88 (73.30)
Blood pressure (mmHg)^α^	
Systolic blood pressure	150.85 ± 20.69
Diastolic blood pressure	88.63 ± 11.51

^α^Values are expressed as averages and standard deviations

^β^Values are expressed in numbers and percentages

**Fig. 1:**
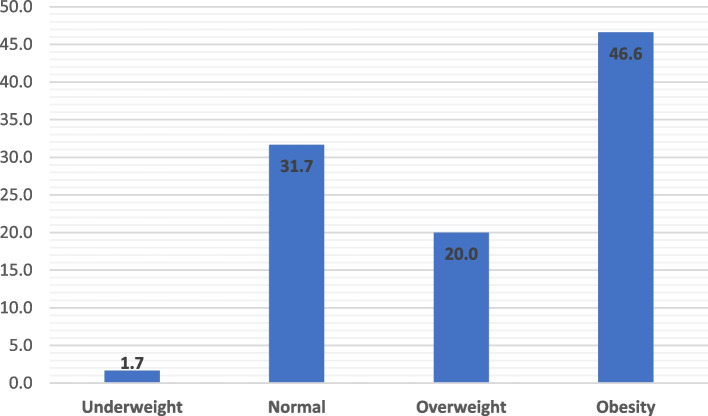
Distribution of hypertensive patients surveyed according to body mass index (BMI) categories. BMI classification was based on the World Health Organization criteria: underweight (<18.5 kg/m²), normal weight (18.5–24.9 kg/m²), overweight (25.0–29.9 kg/m²), and obesity (≥30 kg/m²) [21]

The assessment of the nutritional status of the patients surveyed revealed that only 31.7% (*n* = 38) had a normal BMI and that 46.6% (*n* = 56) suffered from varying degrees of obesity (Fig. 1).

2) Construct validity and reliability of the H-SCALE-M

The psychometric properties of the Moroccan version of the H-SCALE (H-SCALE-M) were assessed through exploratory factor analysis and reliability testing. Construct validity was first examined using exploratory factor analysis. The Kaiser–Meyer–Olkin (KMO) measure of sampling adequacy was 0.68, indicating acceptable suitability for factor analysis, and Bartlett’s test of sphericity was statistically significant (*p* < 0.001), confirming the adequacy of the correlation matrix. Factor extraction using principal axis factoring with Promax rotation identified a six-factor structure consistent with the theoretical dimensions of the original instrument, accounting for 53% of the total variance. Most items showed satisfactory factor loadings (≥ 0.40), supporting the factorial structure of the adapted scale (Table 3).

**Table 3:** Factor structure of the H-SCALE-M (Exploratory factor analysis, *N* = 120)

Domain/Item	Factor loading	Communality
Medication adherence		
Item 1	0.96	0.94
Item 2	0.87	0.77
Item 3	0.99	0.99
Diet quality (DASH diet)		
Item 1	0.73	0.55
Item 2	0.66	0.45
Item 3	0.66	0.46
Item 4	0.41	0.43
Item 5	0.69	0.52
Item 6	0.65	0.49
Item 7	0.43	0.47
Item 8	0.80	0.68
Item 9	0.61	0.46
Item 10	0.59	0.49
Physical activity		
Item 1	0.72	0.53
Item 2	0.71	0.58
Smoking		
Item 1	0.53	0.47
Item 2	0.68	0.52
Weight management		
Item 1	0.67	0.49
Item 2	0.78	0.63
Item 3	0.85	0.76
Item 4	0.60	0.56
Item 5	0.61	0.40
Item 6	0.54	0.51
Item 7	0.74	0.62
Item 8	0.48	0.56
Item 9	0.70	0.57
Item 10	0.41	0.41

H-SCALE-M Moroccan version of Hypertension Self-Care Activity Level effects. Extraction was performed using principal axis factoring with Promax rotation. Only factor loadings ≥ 0.40 are presented

*DASH* Dietary Approaches to Stop Hypertension

The internal consistency of the scale was evaluated using Cronbach’s alpha coefficient and demonstrated acceptable reliability (α = 0.79). All subscales showed satisfactory reliability. However, the items related to alcohol consumption were not included in the psychometric analysis because none of the participants reported alcohol use. Consequently, these items showed no variability and the corresponding subscale could not be evaluated. Test–retest reliability was also considered satisfactory, with an overall intraclass correlation coefficient (ICC) of 0.80, ranging from 0.76 to 0.95 across the different domains of the questionnaire (Table 4).

**Table 4:** Descriptive statistics for the scale (H-SCLAE-M) (*N* = 120)

Sub-scales	Mean ± standarddeviation	Cronbach's alpha	Test/Retest (ICC) CI95%
Adherence to treatment	16.19 ± 7.38	0.91	0.95 (0.92–0.96)
Quality of the DASH diet	41.24 ± 9.11	0.82	0.85 (0.77–0.87)
Physical activity	1.13 ± 1.52	0.71	0.76 (0.67–0.82)
Smoking	1.28 ± 2.90	0.73	0.80 (0.74–0.86)
Weight management	31.45 ± 7.27	0.82	0.84 (0.81–0.89)

*DASH* Dietary Approaches to Stop Hypertension, *H-SCALE-M* Moroccan version of Hypertension Self-Care Activity Level effects, *ICC* Intra-Class Correlation, *IC* Confidence Interval

3) Adherence to Hypertension Self-care Activities

The majority of participants (66.67%; *n* = 80) showed an acceptable level of treatment adherence, with a mean score of 16.19 ± 7.38. In contrast, a significant proportion of patients showed poor adherence to the DASH diet and weight management measures (87.50%; *n* = 105 and 82.50%; *n* = 99, respectively). Self-care activities related to smoking and physical activity were reported less frequently, with mean scores of 1.28 ± 2.90 and 1.13 ± 1.52, respectively (Table 4).

A comparison of extreme groups was used to compare scores between different patient groups according to blood pressure control and gender (Tables 5 and 6). The results revealed a statistically significant difference according to blood pressure control for scores relating to treatment adherence (*p* = 0.01) and weight management (*p* < 0.001). With regard to gender, a significant difference was observed for scores relating to physical activity and tobacco use (*p* = 0.01 and *p* < 0.001, respectively).

**Table 5:** Comparison of scores between the two groups of hypertensive patients according to blood pressure control

	**Blood pressure control**		P Value*
	**Controlled Hypertension (*N* = 32)** **Mean ± Standard deviation**	**Uncontrolled Hypertension (*N* = 88)** **Mean ± Standard deviation**	
Adherence to treatment	20.88 ± 0.71	14.49 ± 7.96	**0.01**
Quality of the DASH diet	39.34 ± 9.66	41.93 ± 8.86	0.36
Physical activity	1.19 ± 1.82	1.10 ± 1.40	0.30
Smoking	0.84 ± 1.93	1.44 ± 3.25	0.71
Weight management	36.19 ± 6.27	29.73 ± 6.86	**< 0.001**

*DASH* Dietary Approaches to Stop Hypertension

*Test t de student; *p* < 0.05 is considered significant

**Table 6:** Comparison of scores between the two groups of hypertensive patients by gender

	**Gender**		P value*
	**Female (*N* = 86) Mean ± Standard deviation**	**Male (*N* = 34) Mean ± Standard deviation**	
Adherence to treatment	16.23 ± 7.30	16.09 ± 7.71	0.92
Quality of the DASH diet	39.92 ± 9.46	44.59 ± 7.27	0.23
Physical activity	0.85 ± 1.27	1.82 ± 1.85	**0.01**
Smoking	0.71 ± 1.80	2.74 ± 4.50	**< 0.001**
Weight management	31.73 ± 7.03	30.74 ± 7.90	0.30

*DASH *Dietary Approaches to Stop Hypertension

*Test t de student; *p* < 0.05 is considered significant

## Discussion

The scale (H-SCALE) designed to assess self-care activities in hypertensive patients has been translated and validated in several languages around the world [24–27], This study is part of this approach and aimed to adapt this scale to the Moroccan dialect and validate it psychometrically.

Overall, the results demonstrated satisfactory reliability and acceptable construct validity of the adapted instrument. The internal consistency of the scale was acceptable and the test–retest reliability indicated good temporal stability. The exploratory factor analysis supported a factorial structure broadly consistent with the theoretical dimensions of the original scale, suggesting that the adapted version adequately captures the main domains of hypertension self-care behaviors. Similar findings have been reported in other validation studies of the H-SCALE conducted in different populations [25–28]. These results confirm the reliability of the scale (H-SCALE-M) for assessing adherence to self-care activities among hypertensive patients who speak Moroccan Arabic dialect.

The scale (H-SCALE) covers all the lifestyle and therapeutic measures that hypertensive patients must follow in order to manage their high blood pressure and prevent the likely complications of uncontrolled hypertension [17], As such, the Moroccan version of H-SCALE is a relevant and accessible tool for healthcare professionals to assess the level of adherence of hypertensive patients to treatment recommendations. All participants responded to items relating to adherence to antihypertensive treatment, following a DASH diet, regular physical activity, smoking cessation, and weight management practices. However, one of the drawbacks of the H-SCALE-M version is that it does not assess alcohol consumption, as none of the participants reported alcohol consumption, the corresponding items showed no variability and were therefore not informative for psychometric analysis. Consequently, the alcohol domain was excluded from the calculation of the overall scale score.

However, alcohol consumption is considered an important factor according to the recommendations of the Seventh Report of the Joint National Committee on Prevention, Detection, Evaluation, and Treatment of High Blood Pressure (JNC 7) [29]. This absence can be explained by the cultural and religious context of Moroccan patients with hypertension. Similar results have been reported in studies conducted in Saudi Arabia [22] and in Iran [23] where items related to alcohol consumption were also excluded from the translated versions for cultural and religious reasons.

Furthermore, the results showed that patients with controlled hypertension had significantly higher scores than those with uncontrolled hypertension, which is consistent with the results of the original study by Warren-Findlow et al. [17]. These results support the ability of the H-SCALE-M to identify areas in which hypertensive patients need to improve their self-management practices. Hypertensive patients with controlled blood pressure demonstrated higher mean scores across several self-care domains compared with those with uncontrolled hypertension. In particular, statistically significant differences were observed for medication adherence and weight management, suggesting that patients who more consistently adhered to antihypertensive treatment and engaged in effective weight-control practices were more likely to achieve adequate blood pressure regulation. These findings are consistent with previous evidence indicating that medication adherence represents one of the most critical determinants of successful hypertension management. Poor adherence to antihypertensive therapy has repeatedly been identified as a major contributor to uncontrolled hypertension and increased cardiovascular risk in clinical practice [30]. Likewise, weight management has been widely recognized as an essential component of hypertension self-care, as excess body weight is strongly associated with elevated blood pressure and adverse cardiovascular outcomes [31] The significantly higher scores observed among patients with controlled hypertension therefore underscore the clinical relevance of these behavioral domains in the effective management of the disease.

The comparison of self-care scores according to gender revealed significant differences in two behavioral domains, physical activity and smoking behavior. Male participants reported higher levels of PA compared with female participants. This finding may partly reflect sociocultural patterns specific to the Moroccan context, where gender roles and social expectations can influence participation in leisure or outdoor PA. In many Moroccan settings, women may face social or environmental constraints that limit their opportunities for structured or recreational PA. In contrast, men may be more likely to engage in occupational or outdoor activities that contribute to higher levels of physical exertion. Similar gender disparities in PA have been reported in studies conducted in North African and Middle Eastern populations, where cultural norms and social roles influence lifestyle behaviors [32].

Similarly, smoking scores were significantly higher among male participants than among female participants. This observation is consistent with epidemiological patterns reported in Morocco and other countries of the Middle East and North Africa region, where tobacco consumption is substantially more prevalent among men than women. Cultural and social norms often discourage smoking among women, while it remains relatively more socially accepted among men. National surveys in Morocco have consistently shown that smoking prevalence among women remains considerably lower than among men, largely due to sociocultural perceptions and gender-related social norms [33]. These contextual factors may therefore contribute to the gender differences observed in smoking-related behaviors in the present study.

In contrast, no statistically significant differences were observed between men and women with regard to medication adherence, adherence to the DASH diet, and weight management behaviors. These findings suggest that certain aspects of hypertension self-care may be relatively similar across genders when patients receive regular follow-up within the healthcare system. In Morocco, the management of chronic diseases such as hypertension is largely supported by primary healthcare services, where patients of both sexes receive comparable medical advice regarding treatment adherence, dietary practices, and lifestyle modifications [34]. Previous studies have shown that adherence to antihypertensive treatment and recommended lifestyle changes is often influenced by factors such as patient education, health literacy, and access to healthcare rather than gender alone [35]. Overall, these findings suggest that while certain self-care behaviors, particularly physical activity and smoking, may be influenced by gender-related sociocultural factors in the Moroccan context, other key aspects of hypertension management appear similar between men and women.

This study has certain limitations. First, Participants were recruited using non-probability sampling from four primary health care centers, which may not fully reflect the diversity of socioeconomic and cultural contexts across the country. Second, the data are based on self-reported information, which may be subject to memory bias or social desirability bias. Third, the lack of assessment of alcohol consumption is a limitation, given its recognized role in uncontrolled blood pressure. For future studies, it would be relevant to consider adapting the scale to other Moroccan dialects, particularly Berber/Amazigh, in order to assess self-management of high blood pressure in populations from different regions of Morocco.

Despite these limitations, this study has several important strengths. To our knowledge, it represents the first study conducted in Morocco to evaluate adherence to hypertension self-care activities using a culturally adapted and psychometrically validated version of the H-SCALE. The study followed a rigorous methodological process of translation, cultural adaptation, and validation, including expert review, face validity assessment, and reliability testing. Furthermore, the use of a dialect-adapted instrument administered through interviewer-assisted questionnaires facilitated comprehension among participants with varying literacy levels, thereby enhancing the accuracy of the collected data. Future research could extend this work by adapting the scale to other Moroccan dialects, particularly Amazigh/Berber languages, in order to evaluate hypertension self-management behaviors in populations from different linguistic and regional backgrounds.

## Conclusion

This study provides evidence supporting the reliability and construct validity of the Moroccan dialect version of the Hypertension Self-Care Activity Level Effects Scale (H-SCALE-M). The adapted instrument demonstrated satisfactory psychometric properties and proved to be a useful tool for assessing adherence to key hypertension self-care behaviors among Moroccan hypertensive patients. The findings also highlight the importance of certain self-management practices, particularly treatment adherence and weight management, in relation to blood pressure control. The availability of a culturally adapted and validated instrument represents an important step toward improving the assessment of self-care behaviors in the Moroccan context. Such tools can facilitate the identification of behavioral gaps in hypertension management and support the development of targeted educational and preventive interventions aimed at improving cardiovascular health outcomes. Further studies involving larger and more diverse populations across different regions of Morocco are recommended to confirm these findings and to explore the broader applicability of the scale in various linguistic and sociocultural settings.

## Data Availability

The datasets generated and/or analyzed in the present study are not publicly accessible owing to ethical considerations. However, they may be obtained from the corresponding author upon reasonable request, subject to approval by the Ethics Committee of the Faculty of Medicine and Pharmacy of Casablanca, Morocco.

## Abbreviations

BMI: Body Mass Index
BPC: Blood Pressure Control
DASH: Dietary Approaches to Stop Hypertension
DBP: Diastolic Blood Pressure
EFA: Exploratory Factor Analysis
H-SCALE: Hypertension Self-Care Activity Level effects
H-SCALE-M: Hypertension Self-Care Activity Level Effects Moroccan version
ICC: Intra-Class Correlation
ICV: Internal content validation
KMO: Kaiser Meyer Olkin
PA: Physical activity
PHCF: Primary Health Care Facilities
SBP: Systolic Blood Pressure
SPSS: Statistical Package for Social Sciences
UBP: Uncontrolled Blood Pressure
WHO: World Health Organization
